# A novel animal model of osteonecrosis of the femoral head induced using a magnetic resonance imaging-guided argon-helium cryotherapy system

**DOI:** 10.3892/etm.2014.1625

**Published:** 2014-03-14

**Authors:** DONG WANG, GUOWEI WANG, MING LIU, LIXIN SUN, WEI ZONG, HONGLEI JIANG, HUAWU ZHANG, HUIBO LI, JIANBAO GONG, SHUI SUN

**Affiliations:** 1 Department of Orthopedics, Provincial Hospital Affiliated to Shandong University, Jinan, Shandong 250021, P.R. China; 2 Department of Orthopedics, Shandong Jiaotong Hospital, Shandong University, Jinan, Shandong 250031, P.R. China; 3 Department of Interventional MRI, Shandong Medical Imaging Research Institute, Shandong University, Jinan, Shandong 250021, P.R. China; 4 Department of Medical Imaging, Provincial Hospital Affiliated to Shandong University, Jinan, Shandong 250021, P.R. China; 5 Department of Cardiology, Provincial Hospital Affiliated to Shandong University, Jinan, Shandong 250021, P.R. China

**Keywords:** osteonecrosis, femoral head, magnetic resonance imaging, cryotherapy, animal model

## Abstract

The aim of the present study was to establish a novel animal model of osteonecrosis of the femoral head (ONFH) using a magnetic resonance imaging (MRI)-guided argon-helium cryotherapy system. A total of 48 rabbits were used to generate the ONFH models. In group I, the left femoral head of the rabbits received two cycles of argon-helium freezing-thawing under MRI guidance, while in group II, the right femoral head of each rabbit received only one cycle of argon-helium freezing-thawing. X-ray and histological examinations were performed. The percentages of lacunae in the femoral heads of group I at weeks 4, 8 and 12 following surgery (49.75±3.17, 62.06±4.12 and 48.25±2.76%, respectively) were higher than those in group II (39.13±4.48, 50.69±3.84 and 37.50±3.86%, respectively). In addition, the percentage of empty lacunae in group I was 62.06% at week 8 following surgery. Therefore, an animal model of ONFH was successfully established using an argon-helium cryotherapy system. The percentage of empty lacunae in group I was higher than that in group II at weeks 4, 8 and 12 after surgery.

## Introduction

Osteonecrosis of the femoral head (ONFH) is an ischemic disease that may result in femoral head collapse. It is caused by multiple factors, such as trauma, alcoholism, long-term use of hormones and Legg-Calve-Perthes disease. The incidence of ONFH in adults is increasing, resulting in significant problems worldwide (1). Although surgical intervention is often used for the treatment of ONFH (2–4), the efficacy is not sufficient for patients. Furthermore, the surgical methods and curative effects are difficult to define as the pathogenesis of ONFH remains unclear (5,6).

Although numerous surgical and non-surgical animal models of ONFH have been established (5–7), there is not a reliable animal model of the early stages of ONFH that may be used for the evaluation of novel therapeutic approaches. Therefore, the aim of the present study was to establish a defined rabbit ONFH model with partial necrosis of the femoral heads using an argon-helium freeze-thaw method under the guidance of magnetic resonance imaging (MRI).

## Materials and methods

### Animal experiments

A total of 48 New Zealand rabbits (weight, 3.50±0.30 kg) were purchased from Xilingjiao Aquaculture Breeding Center (Jinan, China) and used to generate the ONFH models. The rabbits were maintained in standard conditions with free access to food and water. In group I, the left femoral head of every rabbit received two cycles of argon-helium freezing-thawing, while in group II, the right femoral head of each rabbit received only one cycle of argon-helium freezing-thawing. The study was performed in accordance with the Guide for the Care and Use of Laboratory Animals of the National Institutes of Health and the protocol was approved by the Animal Care and Use Committee of Shandong University (Jinan, China).

**Figure 1:**
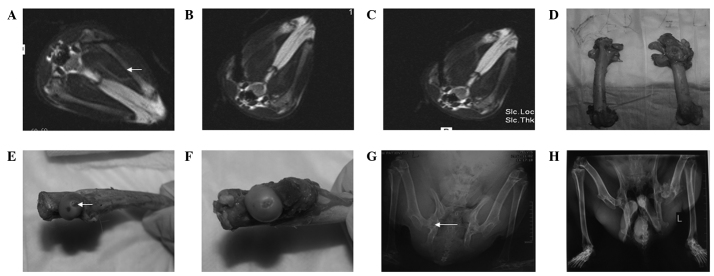
Surgery of the rabbits (groups I and II) was performed in an open 0.23-T MRI system. (A) As guided by MRI, the guide pin was inserted into the bones and located at a position 5 mm below the articular cartilage. (B) In group I rabbits, an ice ball appeared following two freeze-thaw cycles. (C) In group II, one freeze-thaw cycle was performed. (D) At week 4 following the establishment of the model, the femoral head contour was smooth and the cartilage surface was intact, without any defects detected (left, group I left femoral head; right, group II right femoral head). At week 12 following surgery the (E) left femoral head cartilage was defected, as indicated with the arrow, and (F) the right femoral head cartilage was almost integral. (G) At week 8 following surgery, X-ray images showed that the left femoral head had cystic lesions, as indicated with the arrow, while the right femoral head had no abnormal changes. (H) At week 12 following surgery, X-ray images showed that the left femoral head had collapsed, but the right femoral head remained intact. MRI, magnetic resonance imaging.

Using a CRYO-HIT system (Galil Medical Ltd., Yokneam, Israel), the model was established in an MRI interventional unit (Shandong Medical Imaging Research Institute, Jinan, China), which used a 0.23-T open-configuration MRI system mounted with an iPath 200 optical tracking system (Panorama; Philips Medical Systems, Vantaa, Finland). A hole was drilled from the lateral side of the proximal femur into the center of the two femoral heads under MRI guidance. The diameter of the drill track was 2.0 mm and the depth was 5.0 mm under the cartilage (Fig. 1A).

All the femoral heads were evaluated with X-ray scans (X-ray units and DR radiographic systems; General Medical Merate S.p.A, Seriate, Italy) at weeks 4, 8 and 12 following surgery. In total, 16 animals were sacrificed by air embolism at weeks 4, 8 and 12 after surgery and the surface of the cartilage and bone tunnel of the femoral heads were observed.

### Hematoxylin and eosin (HE) histological analyses

A total of 16 rabbits from each group were sacrificed at weeks 4, 8 and 12 following surgery. The two femoral heads of each rabbit were fixed, decalcified, embedded and cut into 5-μm sections. Staining of the samples with HE was then performed. A total of 50 fields were randomly selected and at least 200 lacunae were counted. The percentage of empty lacunae was defined as the ratio of empty lacuna number to the total lacuna count. The mean values of three independent experiments were calculated. The histological images were converted to grayscale images using a computer to calculate the percentage of empty lacunae.

### Statistical analysis

All numerical data are presented as the mean ± SD. The differences between the two groups were calculated using the Student’s t-test. Differences between multiple groups were calculated with one-way analysis of variance (SAS 8.1; SPSS version 17.0, SPSS, Inc., Chicago, IL, USA). Values were considered to indicate a statistically significant difference when P<0.05.

Statistical analyses were performed using the SPSS statistical package, version 17.0 (SPSS, Inc., Chicago, IL, USA). The incidence of femoral collapse in groups I and II were compared using the χ^2^-test. Comparisons of the percentage of empty lacunae between weeks 4, 8 and 12 were performed using an unpaired t-test. P<0.05 was considered to indicate a statistically significant difference.

## Results

### General data of the animals

To establish a novel ONFH animal model using an MRI-guided argon-helium cryotherapy system, 48 rabbits were used. In group I, the left femoral head of every rabbit received two cycles of argon-helium freezing-thawing (Fig. 1B), while in group II, the right femoral head of each rabbit received only one cycle of argon-helium freezing-thawing (Fig. 1C).

In the experiments, none of the rabbits exhibited skin necrosis or infection and there were no mortalities. The femoral head contours of the rabbits in the two groups were smooth and the cartilage surfaces were integral, without any defects detected at week 4 following surgery (Fig. 1D). The femoral head contours of the rabbits in group I became pale, flat and mushroom-shaped, with some bones collapsing at week 12 following surgery (Fig. 1E). Three months after surgery, the right femoral head cartilage was almost intact (Fig. 1F). These results indicated that a novel ONFH animal model using an MRI-guided argon-helium cryotherapy system was successfully established.

### Radiological analysis

To further determine the differences between groups I and II, radiological analyses were performed. In group I, the bone densities of the femoral heads in 10 rabbits were decreased at week 4 following surgery. Cystic changes appeared at week 8 (Fig. 1G) and seven femoral heads were collapsed at week 12 following surgery (Fig. 1H; Table I). In group II, the bone densities of the femoral heads in nine rabbits were reduced at week 4 following surgery. Cystic changes and narrowed hip joint space were observed at week 8 and two femoral heads were collapsed at week 12 (Table I).

### Histological analysis

**Figure 2:**
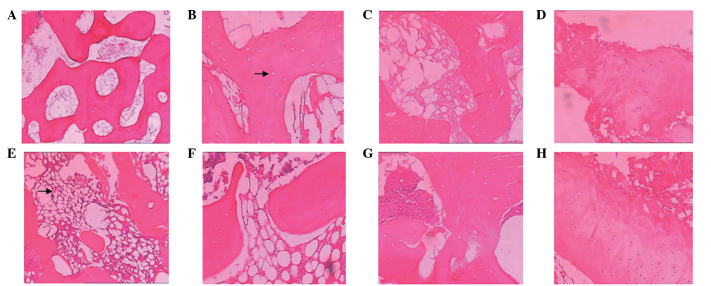
HE staining. (A) A normal femoral head (magnification, ×40). In group I at (B) week 4 following surgery, lacunae (as indicated by the arrow) were observed (magnification, ×100); (C) week 8, the lacunae size increased significantly (magnification, ×100); and (D) week 12, the chondrocytes were disorganized, the articular surface was rough and (E) new bones were formed in the bone necrosis areas, as indicated by the arrow. In group II at (F) week 4 following surgery, the number of cell lacunae was significantly lower than that in group I; (G) week 8, the lacunae cells were identifiable and fibrous tissues had formed; and (H) week 12, cartilage cells were well arranged and no evident collapses were identified in the cartilage surface. HE, hematoxylin and eosin.

HE analyses were performed. A normal femoral head is shown in Fig. 2A and the cases in group I are shown in Fig. 2B–E. At week 4 following the induction of necrosis, a number of osteocytes were necrotic (Fig. 2B), hematopoietic cells were absent and a large number of erythrocytes had died in the marrow cavities. At week 8, the majority of the marrow cavities were filled with fibrous tissue, characterized by a high cellularity with numerous macrophages (Fig. 2C). The chondrocytes were disorganized and the articular surface was rough (Fig. 2D). As observed in an additional group I case, chondrocytes were dispersed (Fig. 2E). Group II cases are shown in Fig. 2F–H. In group II at week 4 following surgery, the percentage of cell lacunae was significantly less when compared with group I (Fig. 2F). In addition, at week 8 following surgery, lacunae cells were easily identifiable and fibrous tissues had formed (Fig. 2G). At week 12 following surgery, the cartilage cells of group II were well arranged and no evident collapses were identified in the cartilage surface (Fig. 2H). As presented in Table II, the percentages of lacunae in the femoral heads of group I at weeks 4, 8 and 12 following surgery (49.75±3.17, 62.06±4.12 and 48.25±2.76%, respectively) were higher than those in group II (39.13±4.48, 50.69±3.84 and 37.50±3.86%, respectively). The percentage of empty lacunae in group I was 62.06% at week 8 following surgery, indicating that bone resorption plays a predominant role. Therefore, the results indicate that the percentage of empty lacunae in group I was higher than that in group II at weeks 4, 8 and 12 following surgery.

## Discussion

Osteonecrosis can be induced by liquid nitrogen and heat (7–11). To the best our knowledge, there are yet to be any studies on the use of cryoablation in establishing an ONFH animal model. The present study used an argon-based system, as discussed in a number of previous studies (12–15), to establish an ONFH animal model. Under the guidance of MRI, the probes can be maintained in the correct position in every femoral head. All the femoral heads of the rabbits used in the present study received precise and well-controlled treatment and two or three freezing cycles led to complete interface sterilization. The differences between a single freezing cycle and two freezing cycles have been demonstrated to be significant (16). The present study divided the rabbits into two groups; group I received two freeze-thaw cycles and group II received one freeze-thaw cycle. The percentage of empty lacunae in group I was higher than that in group II at weeks 4, 8 and 12 following surgery. In addition, a statistically significant difference was observed in the femoral head collapse rates between the two groups. Therefore, the results of the present study indicate that an animal model of ONFH was successfully established using an argon-helium cryotherapy system. Furthermore, MRI-guided argon-helium cryotherapy system may provide animal models of ONFH with high reliability, good repeatability and a precisely controlled necrotic region, which may be of great importance for the study of ONFH

## Figures and Tables

**Table I:** Collapse rates of the femoral heads in groups I and II at week 12 following surgery (n=16).

Group	Collapsed, n	Non-collapsed, n	Collapse rate (%)
I	7	9	43.7^a^
II	2	14	12.5

a P<0.05, vs. group II.

**Table II:** Percentages of lacunae in the femoral heads of groups I and II at weeks 4, 8 and 12 following surgery (n=16).

	Weeks after surgery		
	
Group	4	8	12
I	49.75±3.17	62.06±4.12^a^	48.25±2.76
II	39.13±4.48^b^	50.69±3.84^b^	37.50±3.86^b^

a P<0.05, vs. time points;

b P<0.05, vs. group I.
